# Transcript Patterns of Bovine *CYP21A2* and Its Pseudogene in Adrenal and Ovarian Tissues

**DOI:** 10.3390/genes16111374

**Published:** 2025-11-11

**Authors:** Jakub Wozniak, Monika Stachowiak, Marek Switonski, Joanna Nowacka-Woszuk

**Affiliations:** Department of Genetics and Animal Breeding, Poznan University of Life Sciences, Wolynska 33, 60-637 Poznan, Poland; jakub.wozniak@up.poznan.pl (J.W.); monika.dragan@up.poznan.pl (M.S.); marek.switonski@up.poznan.pl (M.S.)

**Keywords:** *CYP21A2*, pseudogene *CYP21A1P*, transcript variants, cattle, adrenal gland, ovary

## Abstract

Background: The cytochrome P450 family 21 subfamily A member 2 gene (*CYP21A2*) encodes 21-hydroxylase, a key enzyme in adrenal steroid biosynthesis. Despite its physiological importance, the diversity of *CYP21A2* transcript variants and their tissue-specific expression in domestic animals, including cattle, remains largely unexplored. This study aimed to characterize *CYP21A2* transcription in adrenal glands and ovaries and assess the potential transcriptional activity of its pseudogene, *CYP21A1P*. Methods: *CYP21A2* transcription was investigated in adrenal and ovarian tissues of 12 healthy cows using semi-quantitative PCR and Sanger sequencing. Real-time PCR was performed to confirm expression levels. Melting curve analysis and electrophoresis were used to validate distinct amplicons corresponding to different transcript variants. Extended amplicons were sequenced to identify transcripts corresponding to reference sequences and potential pseudogene products. Results: A single transcript variant (NM_001013596.1) was consistently detected in adrenal glands, whereas ovaries expressed two variants: NM_001013596.1 and XM_024983378.2. Semi-quantitative analysis showed significantly higher *CYP21A2* expression in adrenal glands compared to ovaries (*p* < 0.01). In ovarian samples, the NM_001013596.1 variant was more abundant than the XM_024983378.2 (*p* < 0.01). Sanger sequencing revealed two products matching *CYP21A2* reference transcripts and an additional, longer product containing sequence motifs specific to the pseudogene *CYP21A1P*, indicating its transcriptional activity. Conclusions: These results provide the first evidence of tissue-specific expression and differential abundance of *CYP21A2* transcript variants in cattle and suggest the transcription of the *CYP21A1P* pseudogene. The findings reveal the complexity of *CYP21A2* expression in steroidogenic tissues and suggest potential regulatory roles for transcript and pseudogene variants in bovine physiology.

## 1. Introduction

The cytochrome P450 21A2 (*CYP21A2*) gene encodes the steroid 21-hydroxylase enzyme, which is responsible for hydroxylating progesterone and 17-hydroxyprogesterone, as well as reducing molecular oxygen, as shown in humans [1,2].

Near the locus of *CYP21A2*, its pseudogene (*CYP21A1P*) is located, and in humans, they share 98% nucleotide sequence similarity in exons and 96% similarity in introns [3]. It is assumed that due to a very high sequence similarity, the majority of the identified variants arose from gene conversion events or non-homologous recombination [1,4,5,6]. It is also known that approx. 1441 human pseudogenes can be transcribed [7]. In humans, the transcription of the *CYP21A2* pseudogene was confirmed using the Northern blot method [8]. To the best of our knowledge, no transcriptomic or genomic studies have demonstrated *CYP21A1P* expression in cattle; therefore, our results provide the first evidence addressing this unexplored aspect of the bovine CYP21 locus.

Molecular knowledge on bovine *CYP21A2* and *CYP21A1P* is scarce. It is known that both loci, similarly to the human genome, are located within the class III region of the major histocompatibility complex [9]. According to the NCBI Gene database, there are several transcript variants of bovine *CYP21A2* (see NCBI Gene: *CYP21A2*; https://www.ncbi.nlm.nih.gov/gene/281741, accessed on 10 February 2023). In livestock, promoter polymorphisms of *CYP21* have been investigated in relation to milk production and growth traits, although results have been inconsistent, and in some studies, the nomenclature did not clearly specify whether the variants were located in the functional gene or in the pseudogene [10,11,12]. While some studies have reported *CYP21A2* expression in tissues beyond the adrenal gland—for instance, in the ovary during early pregnancy [13]—comprehensive analyses of its expression across a broader range of tissues, along with investigations of transcript variability, remain lacking.

Mutations in *CYP21A2* are well-known causes of human autosomal recessive disorders of adrenal steroidogenesis, known as congenital adrenal hyperplasia (CAH), resulting from 21-hydroxylase deficiency [14]. The wide phenotypic spectrum, from classic to non-classical forms, illustrates how different mutations affecting CYP21A2 activity can lead to variable clinical outcomes. In a study by Carvalho et al. [15], it was already shown that similarity between the *CYP21A2* gene and its pseudogene (*CYP21A1P*) predisposes the region to genetic recombination events, leading to the transfer of pathogenic variants from the pseudogene to the functional gene. Approximately 75% of deleterious variants in the *CYP21A2* gene result from gene conversion during mitosis, where segments of DNA are transferred from the pseudogene to the active gene. Around 20% of mutations arise due to unequal crossing over during meiosis, leading to duplications or deletions within the *CYP21A2* gene [15]. Although this wealth of insight exists in human endocrinology, no analogous clinical or molecular evidence for *CYP21A2*-linked CAH has been reported in cattle.

Usually, the CAH is associated with disorders of sex development (DSDs) in girls, and it is the most common monogenic XX DSD in humans [16]. In contrast, individuals with the non-classical type typically develop symptoms related to androgen excess, and due to partial retention of 21-hydroxylase activity, they usually exhibit a mild and variable phenotype [1,14]. Studies of DSD in cattle have been mainly focused on freemartinism in heifers, associated with leukocyte XX/XY chimerism, originating from heterosexual twins [17]. On the contrary, XX DSD in cattle was reported very rarely [18,19], in spite of the fact that this form of DSD is not rare in goats, pigs, horses, and dogs [20]. *CYP21A2* has not yet been studied in domestic animals affected with DSD.

Given the essential role of *CYP21A2* in steroid hormone biosynthesis, investigating its expression in cattle may yield valuable insights into adrenal and gonadal steroidogenesis in livestock. Steroid 21-hydroxylase (the CYP21A2 enzyme) mediates key steps in cortisol and aldosterone synthesis, and alterations in its function are central to CAH in humans [21]. Comparative analyses of *CYP21A2* expression patterns across species can help elucidate conserved regulatory mechanisms in reproductive endocrinology. In farm animals, adrenal and ovarian-derived steroid hormones (e.g., cortisol and progesterone) are fundamental regulators of reproductive performance, fertility, and adaptation to stress. Thus, dysregulation of CYP21A2 activity may influence both reproductive disorders and production efficiency. Indeed, stress–endocrine interactions have been shown to markedly impact reproductive efficiency in cattle via endocrine, paracrine, and neural pathways [22].

The aim of this study was to identify the *CYP21A2* transcript variants in cow adrenal glands and ovaries, as well as to detect the potential transcription of its pseudogene (*CYP21A2*).

## 2. Materials and Methods

### 2.1. Ethics Statement

The samples in this project were collected during routine commercial slaughter. This case is covered by Polish law and did not require the approval of the local Bioethical Commission for Animal Care and Use in Poznan, Poland. Animals were not subjected to any experimental procedures prior to slaughter, and tissue sampling was performed exclusively from post-mortem by-products, in accordance with animal welfare regulations (including Directive 2010/63/EU).

### 2.2. Animals and Sample Collection

Ovarian (*n* = 6) and adrenal gland (*n* = 6) samples were collected from 12 adult Holstein-Friesian cows during routine commercial slaughter at an abattoir located in the Wielkopolska Province, Poland. Animals were euthanized using percussive stunning (headshot), which involved a single impact to the head with a captive bolt pistol, resulting in immediate loss of consciousness without the use of any chemicals. Following euthanasia, tissue samples (post-mortem by-products) were collected, rapidly frozen in liquid nitrogen, and stored at −80 °C for further RNA isolation.

### 2.3. RNA Extraction and cDNA Synthesis

Total RNA was isolated using the RNeasy Fibrous Tissue Mini Kit (Qiagen, Germantown, MD, USA) from 12 cows (6 samples of ovaries and 6 samples of adrenal gland). Approximately 30 mg of tissue from each sample was homogenized in the RTL buffer provided with the kit, utilizing the TissueLyser LT (Qiagen, Germantown, MD, USA). Subsequent steps followed the manufacturer’s protocol (including the DNA-se digestion step). RNA concentration and purity were measured with a NanoDrop ND-2000 spectrophotometer (Thermo Fisher Scientific, Waltham, MA, USA), based on absorbance at 260/280 nm (Supplementary Materials, Table S1).

For cDNA synthesis, roughly 1 µg of RNA per sample was reverse-transcribed using the Transcriptor First Strand cDNA Synthesis Kit (Roche, Mannheim, Germany) in a final reaction volume of 20 µL, according to the manufacturer’s instructions. The obtained cDNA was evaluated again with the NanoDrop ND-2000 spectrophotometer and diluted with nuclease-free water.

### 2.4. Semi-Quantitative Real-Time PCR

**Figure 1:**
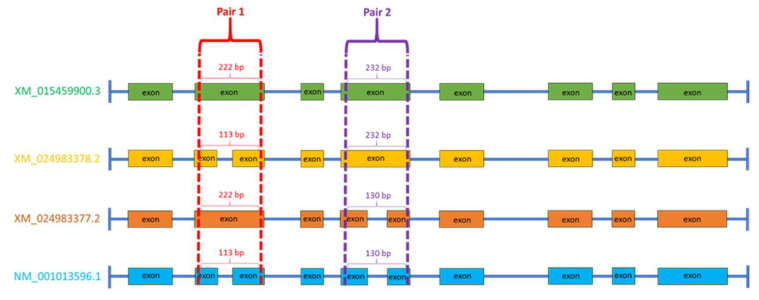
Schematic illustration of PCR product sizes of *CYP21A2* amplified with primer pairs 1 and 2 for different transcript variants (amplicon size determined on cDNA sequence of each transcript—without introns). Primer pair 1 amplifies products of 222 bp for variants XM_015459900.3 and XM_024983377.2 and shorter products of 113 bp for XM_024983378.2 and NM_001013596.1. Primer pair 2 produces 232 bp fragments for XM_015459900.3 and XM_024983378.2 and shorter 130 bp fragments for XM_024983377.2 and NM_001013596.1.

cDNA served as a template for real-time PCR performed on the LightCycler^®^ 480 II system (Roche, Mannheim, Germany). The expression level of the *CYP21A2* gene was assessed in both ovarian and adrenal gland tissues. Primers were designed using the Primer3Plus tool (https://www.primer3plus.com, accessed on 1 March 2023). To verify the presence of specific transcript variants, the identity of semi-qPCR products was assessed by combining melting curve analysis and agarose gel electrophoresis. To distinguish between four annotated transcript variants of the *CYP21A2* gene (XM_015459900.3, XM_024983378.2, XM_024983377.2, and NM_001013596.1), two primer pairs were designed: pair 1 and pair 2 (Supplementary Material, Table S2). Pair 1 generates PCR products of the same length for variants XM_015459900.3 and XM_024983377.2 (222 bp) and a shorter product of identical length for XM_024983378.2 and NM_001013596.1 (113 bp). Pair 2 produces PCR products of the same length for XM_015459900.3 and XM_024983378.2 (232 bp) and a shorter product of identical length for XM_024983377.2 and NM_001013596.1 (130 bp), as shown in Figure 1. By combining results from both primer pairs, it was possible to reliably discriminate among all four transcript variants based on product size and melting temperature profiles.

All semi-qPCR reactions were conducted in duplicate, using the LightCycler^®^ 480 SYBR Green I Master mix (Roche, Mannheim, Germany), with a total reaction volume of 10 μL per well, following the manufacturer’s instructions. The thermal cycling conditions included an initial denaturation step at 95 °C for 10 min, followed by 45 amplification cycles comprising denaturation at 95 °C for 10 s, primer annealing at 62 °C for 5 s, and extension at 72 °C for 5 s. To confirm the specificity of the amplified products, a melting curve analysis was performed after each semi-qPCR run. Next, the amplicon lengths were evaluated using 2% agarose gel electrophoresis, with visualization on the Chemi Doc MP Imagine System (Bio-Rad, Hercules, CA, USA).

The relative semi-quantitative transcript levels were quantified based on band insensitivity measurements (Image Lab, version 6.0.1 build 34, Bio-Rad, Hercules, CA, USA) after agarose electrophoresis. The results from each *CYP21A2* transcript band were normalized to the reference *TATA-Box Binding Protein* gene. The obtained mean values were statistically analyzed. The densitometry values of amplicons are presented in Supplementary Materials, Table S3.

### 2.5. PCR Amplification and Gel Purification of cDNA Fragments

Since pairs 1 and 2 used for semi-qPCR did not distinguish between amplicons from *CYP21A2* and its pseudogene, the additional primer pair was designed to amplify a longer fragment spanning exons 1-3 of the gene and its pseudogene (Supplementary Material, Table S2). The expected PCR product lengths were as follows: 667 bp (for XM_015459900.3 and XM_024983377.2), 559 bp (for XM_024983378.2 and NM_001013596.1), and 762 bp for the pseudogene. Polymerase chain reactions were carried out using a Bio-Rad thermocycler. Each reaction mixture (20 µL) contained 10 ng of cDNA, 10× reaction buffer B (EURx), a mix of ultrapure dNTPs (1.25 nM each, EURx), 5 nM of each primer, 1 unit of Taq DNA Polymerase (EURx, Gdansk, Poland), and nuclease-free water. The PCR program included an initial denaturation at 95 °C for 10 min, followed by 38 cycles consisting of denaturation at 95 °C for 40 s, primer annealing at 63 °C, and elongation at 72 °C for 40 s. The final extension was performed at 72 °C for 10 min, after which samples were held at 4 °C. Negative controls without the cDNA template were included.

The amplicon lengths were evaluated using 2% agarose gel electrophoresis, with visualization on the Chemi Doc MP Imagine System (Bio-Rad, Hercules, CA, USA).

### 2.6. Sanger Sequencing Analysis of cDNA

Individual PCR products (three separate bands) were cut out of the agarose gel and purified with the GeneJET Gel Extraction Kit (Thermo Fisher Scientific, Waltham, MA, USA), followed by sequencing reactions using the BigDye Terminator v3.1 Cycle Sequencing Kit (Life Technologies, Waltham, MA, USA) according to the manufacturer’s guidelines. After sequencing amplification, products were purified through Sephadex G50 columns (Sigma, Darmstadt, Germany). Capillary electrophoresis was conducted using the Genetic Analyzer 3500 (Applied Biosystems, Waltham, MA, USA). Sequencing data were analyzed with DNASTAR software (DNASTAR, Madison, WI, USA), and the obtained sequences were aligned to reference sequences from the GenBank database: NC_037350 for *CYP21A2* and XR_003032199.2 for *CYP21A1P.*

### 2.7. Statistical Analysis

Statistical analysis was carried out in the R software using the stats package [23]. The Shapiro–Wilk test was used to test the normality of the data. Subsequently, a nonparametric two-tailed Mann–Whitney U-test was performed for results from semi-qPCR.

## 3. Results

### 3.1. Semi-Quantitative PCR Results

**Figure 2:**
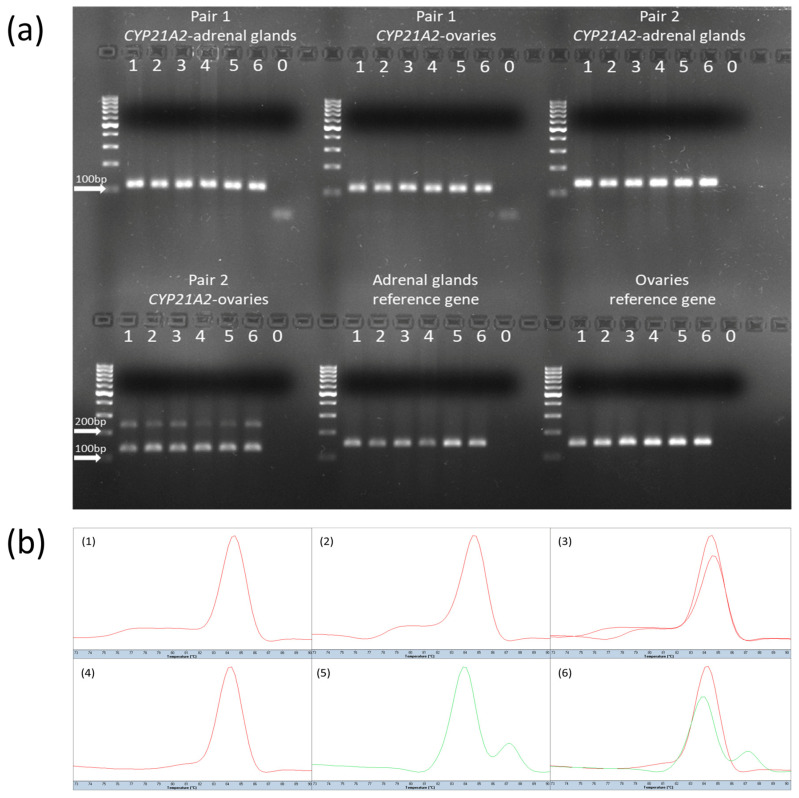
Verification of *CYP21A2* transcript variant expression using electrophoresis and melting curve analysis. (**a**) Representative agarose gel electrophoresis showing qPCR products amplified with primer pair 1 and pair 2 in cDNA from adrenal glands and ovaries. Reference gene amplification is shown in the bottom-middle and -right panels. Each lane represents a separate biological replicate (lines 1–6); the final, seventh lane (0) in each group is a no-template control. Arrows indicate 100 or 200 bp bands on a 100 bp DNA ladder (Thermo Fisher Scientific). (**b**) Melting curve profiles of the qPCR products. (**1**) Pair 1—adrenal glands; (**2**) pair 1—ovaries; (**3**) pair 1—overlaid curves (adrenal glands and ovaries); (**4**) pair 2—adrenal glands; (**5**) pair 2—ovaries; (**6**) pair 2—overlaid curves (adrenal glands and ovaries).

Real-time PCR confirmed the expression of *CYP21A2* transcripts in both adrenal gland and ovarian tissue samples. In all adrenal gland samples, a single transcript variant was detected, corresponding to the NM_001013596.1 transcript, as indicated by the consistent presence of the 113 bp (pair 1) and 130 bp (pair 2) products, along with uniform melting curve profiles, as shown in Figure 2. In ovarian samples, two transcript variants were observed: NM_001013596.1 and XM_024983378.2. These were confirmed by the presence of 113 bp products from pair 1, 130 bp and 232 bp products from pair 2, and distinct double peaks in the melting curve analysis, as shown in Figure 2.

**Figure 3:**
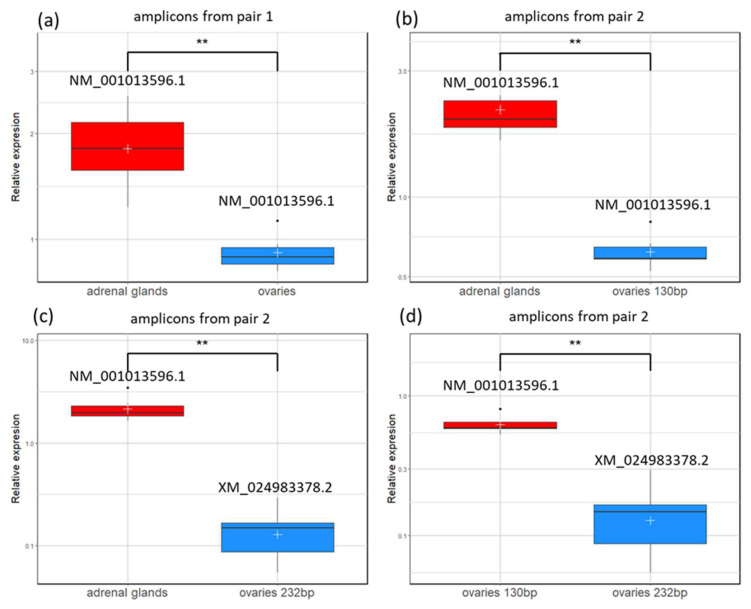
Boxplots presenting expression levels of *CYP21A2* transcript variants detected by semi-qPCR with different primer pairs. (**a**) Pair 1—comparison between adrenal glands and ovaries for the NM_001013596.1 transcript variant; (**b**) pair 2—comparison between shorter amplicons (130 bp) detected in adrenal glands and ovaries for the NM_001013596.1 transcript variant; (**c**) pair 2—comparison between the NM_001013596.1 transcript variant in adrenal glands and the XM_024983378.2 transcript variant in ovaries (232 bp); and (**d**) pair 2—comparison between NM_001013596.1 and XM_024983378.2 variants in ovaries. The red color indicates adrenal samples (or shorter transcript variants in the case of comparison within ovarian samples), and blue indicates ovarian samples (or longer transcript variants in the case of comparison within ovarian samples). The vertical black lines crossing the boxes show medians. The lines below and above the rectangles indicate the maximum and minimum values, the black dots positioned beneath and above the boxes represent outliers, and white crosses show mean values. Asterisks indicate statistical significance between compared samples: ** *p* < 0.01.

Semi-quantitative analysis revealed significant differences in the *CYP21A2* transcript level in the studied samples (Figure 3). For the NM_001013596.1 transcript, a higher level was observed in adrenal glands than in ovaries, with no difference if pair 1 or 2 was used for amplification (*p* < 0.01). The transcript level of NM_001013596.1 in adrenal glands was also significantly higher than the level of the longer product (232 bp) representing the XM_024983378.2 transcript amplified from ovarian tissue (*p* < 0.01).

Further analysis of ovarian samples indicated that the shorter product (NM_001013596.1) consistently had a higher level than XM_024983378.2 (*p* < 0.01), as shown in Figure 3.

### 3.2. Sanger Sequencing Analysis

**Figure 4:**
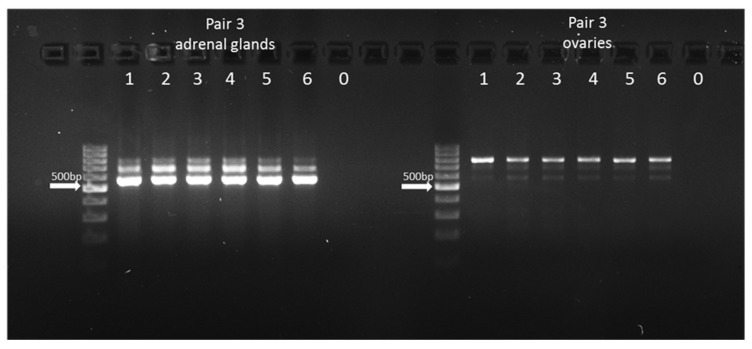
Electrophoresis of cDNA-based PCR amplification from adrenal glands and ovaries (lines 1–6—studied samples; 0—no-template control). Arrows indicate 500 bp on a 100 bp DNA ladder (Thermo Fisher Scientific). Three amplicons of 762, 667, and 559 bp were identified.

Since the same amplicon size product (113 bp for pair 1 and 130 bp for pair 2) could potentially originate from a pseudogene, the additional primer pair (pair 3) was used for cDNA amplification. This allowed us to identify three PCR products in both studied tissues. Two amplicons (559 and 667 bp) represented the *CYP21A2* transcripts. It was assumed that a longer amplicon (762 bp) represents the *CYP21A1P* pseudogene transcript (Figure 4). Sanger sequencing of all three amplicons revealed that the 559 bp product showed complete concordance with the reference transcript sequences, with no SNPs or deletions detected in any of the analyzed samples. The 667 bp product also showed complete identity with the reference; however, in some samples, low-intensity secondary peaks were observed at positions corresponding to known differences between bovine *CYP21A2* and *CYP21A1P* (positions 23:27330052G, 23:27330003C, 23:27329990C, and 23:27329984C in *CYP21A2* and 23:27368802A, 23:27368753T, 23:27368740G, and 23:27368734_27368733delC in *CYP21A1P*). Sequencing of the 762 bp amplicon revealed predominantly pseudogene-specific sequence features (Supplementary Materials, Figure S1). The sequence alignment of a *CYP21A2* gene fragment and its *CYP21A1P* pseudogene with nucleotide differences is presented in Supplementary Materials, Figure S2.

## 4. Discussion

Our results demonstrate that *CYP21A2* is actively transcribed in both bovine tissues—adrenal glands and ovaries—with a predominant presence of NM_001013596.1 in adrenal tissue and a dual expression of NM_001013596.1 and XM_024983378.2 transcripts in ovarian samples. In addition, the detection of a pseudogene-derived transcript (762 bp) suggests an additional layer of regulation, for instance, by acting as a competing endogenous RNA that modulates *CYP21A2* transcript stability, or by serving as a source of small regulatory RNAs. Ovarian expression of *CYP21A2* may have been underestimated, yet evidence indicates that *CYP21A2* is expressed in granulosa cells and in the corpus luteum (CL), highlighting its role not only as a key adrenal enzyme but also as a potentially important player in local ovarian steroid metabolism. In particular, *CYP21A2* activity may contribute to mineralocorticoid synthesis, which has been linked to follicular maturation and CL function [24]. Moreover, decreased expression of this gene was observed in CL of cows during early pregnancy [13]. The differences observed here in the *CYP21A2* expression levels between the adrenal gland and ovaries are in agreement with basic knowledge that its high expression in the adrenal cortex reflects the need for the production of corticosteroids, which are essential for electrolyte balance, blood pressure regulation, and stress responses. In the ovary, *CYP21A2* expression is much lower but important for local steroid metabolism [24].

The suggestive transcriptional activity of the *CYP21A1P* pseudogene is an interesting finding. While pseudogenes were long considered non-functional genome elements, recent evidence supports their regulatory potential through coding-independent mechanisms. For instance, the bovine aromatase pseudogene *CYP19P1* is expressed in the placenta and has been suggested to modulate *CYP19A1* expression [25]. By analogy, *CYP21A1P* may exert a similar modulatory effect on *CYP21A2*, particularly when co-expressed in steroidogenic tissues.

In humans, *CYP21A1P* shares 98% sequence identity with *CYP21A2* and is transcribed in the adrenal gland, albeit at lower levels (10–20% compared with *CYP21A2*) [8]. While such data have not yet been confirmed in cattle, the detected 762 bp amplicon exhibited pseudogene-specific features, suggesting its transcriptional activity. Moreover, the observed differences in band intensity between studied tissues indicate relatively higher amplification from *CYP21A2* gene transcripts than from its *CYP21A1P* pseudogene in the adrenal gland, and the reverse situation is found in the ovaries. It should be noted, however, that the appearance of two unexpected bands (559 and 667 bp—potential transcript variants of *CYP21A2*) in adrenal samples contrasts with the pattern obtained for the shorter amplicons used for the semi-quantitative approach, where only one transcript variant was identified. This discrepancy may result from higher primer specificity and amplification efficiency for shorter fragments, suggesting that in the bovine adrenal gland, only one transcript (NM_001013596.1) is predominantly expressed. As a limitation of this work, we need to point out that the observed double peaks at four specific positions may reflect the presence of mixed PCR products derived from both the gene and the pseudogene, which indicates technical difficulties with clear separation and purification after agarose gel electrophoresis. However, the presence of the longest PCR product, confirmed by the Sanger method, being sequence-specific for *CYP21A1P*, suggests the transcriptional activity of this pseudogene. Nevertheless, several methodological limitations must be acknowledged. The semi-quantitative nature of the PCR applied in this study only allows for approximate comparisons of transcript abundance and does not reflect absolute expression levels. Therefore, differences in band intensity should be interpreted with caution, as they may also reflect variations in amplification efficiency or template composition. Additionally, due to the high sequence similarity between *CYP21A2* and its pseudogene *CYP21A1P*, the possibility of cross-amplification cannot be excluded, highlighting technical challenges related to achieving complete separation and purification of closely related products after agarose gel electrophoresis. Despite these limitations, the longest PCR product exhibited a sequence confirmed by Sanger sequencing as *CYP21A1P*-specific, providing molecular evidence that this pseudogene may be transcriptionally active in cattle.

Pseudogenes may influence their parental gene by acting as RNA decoys, modulating transcript stability, or serving as competing endogenous RNAs. In humans, pseudogene-derived mutations transferred to *CYP21A2* by gene conversion or promoter alterations can result in non-classical congenital adrenal hyperplasia (NC-CAH), with measurable effects on transcriptional efficiency [26]. Thus, it is plausible that bovine *CYP21A1P* transcription contributes to regulatory crosstalk with *CYP21A2*. From an evolutionary perspective, the persistence of pseudogene transcription suggests that these elements may provide selective advantages through regulatory functions rather than protein-coding capacity. Although initially regarded as non-functional by-products of genome evolution, increasing evidence indicates that pseudogenes can contribute to gene regulatory networks in diverse ways. For example, some pseudogene transcripts have been shown to act as competing endogenous RNAs (ceRNAs), serving as decoys for microRNAs and thereby modulating the expression of their parental genes. A well-known case is the pseudogene *PTENP1*, which regulates the tumor suppressor *PTEN* by sequestering specific miRNAs such as miR-499-5p. This mechanism shows how pseudogene-derived transcripts, even if noncoding, may exert significant post-transcriptional regulatory effects [27]. In cattle, if *CYP21A1P* is indeed transcribed, analogous ceRNA-like functions could be considered, whereby pseudogene transcripts influence *CYP21A2* or other components of steroidogenic pathways. Such a role has been proposed for pseudogenes in other systems, for example, in breast cancer [28]. Although speculative at this stage, such regulatory interactions could add an additional layer of complexity to the control of steroid biosynthesis and reproductive physiology in cattle. Others give rise to small interfering RNAs that participate in post-transcriptional silencing, or they may influence chromatin organization and transcriptional activity. The retention of pseudogenes in mammalian genomes is therefore not simply a reflection of neutral drift but may represent adaptive value associated with their noncoding regulatory roles [29]. Our identification of pseudogene transcripts in the bovine tissues suggests a possible role in diversifying local steroid metabolism.

## 5. Conclusions

This study provides novel insights into the complexity of *CYP21A2* transcription in cattle, revealing both tissue-specific transcript variant expression and preliminary evidence that *CYP21A1P* may be transcribed. These findings raise the possibility that pseudogene-derived transcripts could play a role in regulatory processes in bovine steroidogenic tissues. Future work should further clarify their occurrence, functional relevance, and evolutionary significance, potentially linking pseudogene transcription to aspects of reproductive physiology and health in domestic animals.

## Data Availability

The original contributions presented in this study are included in the article/Supplementary Materials. Further inquiries can be directed to the corresponding author.

## Supplementary Materials

The following supporting information can be downloaded at https://www.mdpi.com/article/10.3390/genes16111374/s1: Table S1. RNA concentration (ng/µL) and purity (A260/280nm) of adrenal gland and ovarian samples. Table S2. Primers used in qPCR and Sanger sequencing. Table S3. Densitometric values of *CYP21A2* amplicons obtained by semi-quantitative PCR. Background-corrected integrated density (Adj. Total Band Volume) is shown for each band amplified with primer pair 1 or 2 and reference TATA_BOX from adrenal gland and ovarian tissue. Figure S1. Sanger sequencing chromatograms of cDNA-derived PCR products (559 bp, 667 bp, and 762 bp) for four identified variant positions (a–d). Each panel (a–d) corresponds to a distinct variant and shows chromatograms for the 559 bp product (top), 667 bp product (middle), and 762 bp product (bottom). Blue rectangles indicate the positions of the analyzed variants. In the 667 bp and 762 bp products, secondary or mixed peaks are visible at several positions, reflecting sequence similarity with the *CYP21A1P* pseudogene. Figure S2. Alignment of a partial exon 1 sequence of the *CYP21A2* and corresponding pseudogene fragment with 4 differences found in this study (indicated by red arrows).

## Abbreviations

The following abbreviations are used in this manuscript:

DSDs	Disorders of sex development
cDNA	Complementary DNA
semi-qPCR	Semi-quantitative real-time PCR
CL	Corpus luteum
ceRNAs	Competing endogenous RNAs

## References

[1] Haider S., Islam B., D’Atri V., Sgobba M., Poojari C., Sun L., Yuen T., Zaidi M., New M.I. (2013). Structure-phenotype correlation of human CYP21A2 mutations in congenital adrenal hyperplasia. Proc. Natl. Acad. Sci. USA.

[2] White P.C., Speiser P.W. (2000). Congenital adrenal hyperplasia due to 21-hydroxylase deficiency. Endocr. Rev..

[3] White P.C., New M.I., Dupont B. (1986). Structure of human steroid 21-hydroxylase genes. Proc. Natl. Acad. Sci. USA.

[4] Donohoue P.A., van Dop C., McLean R.H., White P.C., Jospe N., Migeon C.J. (1986). Gene conversion in salt-losing congenital adrenal hyperplasia with absent complement C4B protein. J. Clin. Endocrinol. Metab..

[5] Higashi Y., Tanae A., Inoue H., Fujii-Kuriyama Y. (1988). Evidence for frequent gene conversion in the steroid 21-hydroxylase P-450(C21) gene: Implications for steroid 21-hydroxylase deficiency. Am. J. Hum. Genet..

[6] Concolino P., Falhammar H. (2025). Genetics in Congenital Adrenal Hyperplasia Due to 21-Hydroxylase Deficiency and Clinical Implications. J. Endocr. Soc..

[7] Sisu C., Pei B., Leng J., Frankish A., Zhang Y., Balasubramanian S., Harte R., Wang D., Rutenberg-Schoenberg M., Clark W. (2014). Comparative analysis of pseudogenes across three phyla. Proc. Natl. Acad. Sci. USA.

[8] Bristow J., Gitelman S.E., Tee M.K., Staels B., Miller W.L. (1993). Abundant adrenal-specific transcription of the human P450c21A “pseudogene”. J. Biol. Chem..

[9] Gelhaus A., Hess M., Förster B., Goldammer T., Schwerin M., Horstmann R.D. (2006). YAC/BAC contig spanning the MHC class III region of cattle. Cytogenet. Genome Res..

[10] Martins da Silva A., Rios A.F., Ramos E.S., Lôbo R.B., Oliveira H.N., de Freitas M.A. (2011). Association between IGF2 and CYP21 gene polymorphisms and characteristics of economic interest in Nellore cattle. Genet. Mol. Res..

[11] Hassooni H., Shadood S. (2023). Relationship between the CYP21 marker and some productive and physiological traits of Holstein-Friesian cows. IOP Conf. Ser. Earth Environ. Sci..

[12] Jędrzejczak M., Grzesiak W., Szatkowska I., Dybus A., Muszyńska M., Zaborski D. (2011). Association between polymorphisms of CYP19, CYP21, and ER1 genes and milk production traits in Black-and-White cattle. Turk. J. Vet. Anim. Sci..

[13] Sakumoto R., Hayashi K.G., Hosoe M., Iga K., Kizaki K. (2020). Pregnancy-associated changes of peroxisome proliferator-activated receptor delta (PPARD) and cytochrome P450 family 21 subfamily A member 2 (CYP21A2) expression in the bovine corpus luteum. J. Reprod. Dev..

[14] Simonetti L., Bruque C.D., Fernández C.S., Benavides-Mori B., Delea M., Kolomenski J.E., Espeche L.D., Buzzalino N.D., Nadra A.D., Dain L. (2018). CYP21A2 mutation update: Comprehensive analysis of databases and published genetic variants. Hum. Mutat..

[15] Carvalho B., Marques C.J., Santos-Silva R., Fontoura M., Carvalho D., Carvalho F. (2021). Congenital Adrenal Hyperplasia Due to 21-Hydroxylase Deficiency: An Update on Genetic Analysis of CYP21A2 Gene. Exp. Clin. Endocrinol. Diabetes..

[16] Gusmano C., Cannarella R., Crafa A., Barbagallo F., La Vignera S., Condorelli R.A., Calogero A.E. (2023). Congenital adrenal hyperplasia, disorders of sex development, and infertility in patients with POR gene pathogenic variants: A systematic review of the literature. J. Endocrinol. Investig..

[17] Iannuzzi A., Parma P., Iannuzzi L. (2021). Chromosome Abnormalities and Fertility in Domestic Bovids: A Review. Animals.

[18] Bresciani C., Parma P., De Lorenzi L., Bigliardi E., Cantoni A.M., Morini G., Parmigiani E. (2015). A very rare clinical case of a holstein heifer with two vulvae and a scrotum. Sex. Dev..

[19] St-Jean G., Charreton-Sanford V., Pesant M.J., Zamberlam G., Boyer A., Beaudoin G., Gagnon C.A. (2024). Sex cord-stromal (granulosa cell) tumor in an ovotestis from a cow. Can. Vet. J..

[20] Parma P., Veyrunes F., Pailhoux E. (2016). Sex Reversal in Non-Human Placental Mammals. Sex. Dev..

[21] Turcu A.F., Auchus R.J. (2015). Adrenal steroidogenesis and congenital adrenal hyperplasia. Endocrinol. Metab. Clin. N. Am..

[22] Fernandez-Novo A., Pérez-Garnelo S.S., Villagrá A., Pérez-Villalobos N., Astiz S. (2020). The Effect of Stress on Reproduction and Reproductive Technologies in Beef Cattle-A Review. Animals.

[23] R Core Team (2023). R: A Language and Environment for Statistical Computing.

[24] Mukangwa M., Takizawa K., Aoki Y., Hamano S., Tetsuka M. (2020). Expression of genes encoding mineralocorticoid biosynthetic enzymes and the mineralocorticoid receptor, and levels of mineralocorticoids in the bovine follicle and corpus luteum. J. Reprod. Dev..

[25] Chwalisz M., Fürbass R. (2014). Evaluation of coding-independent functions of the transcribed bovine aromatase pseudogene CYP19P1. BMC Res. Notes.

[26] Araújo R.S., Mendonca B.B., Barbosa A.S., Lin C.J., Marcondes J.A., Billerbeck A.E., Bachega T.A. (2007). Microconversion between CYP21A2 and CYP21A1P promoter regions causes the nonclassical form of 21-hydroxylase deficiency. J. Clin. Endocrinol. Metab..

[27] Wang L., Zhang N., Wang Z., Ai D.M., Cao Z.Y., Pan H.P. (2016). Pseudogene PTENP1 Functions as a Competing Endogenous RNA (ceRNA) to Regulate PTEN Expression by Sponging miR-499-5p. Biochemistry.

[28] Mohebifar H., Sabbaghian A., Farazmandfar T., Golalipour M. (2023). Construction and analysis of pseudogene-related ceRNA network in breast cancer. Sci. Rep..

[29] Tang J.S., Yang M.Y., Li Y. (2015). Functional roles of pseudogenes in cancers. Yi Chuan.

