# Supplementary material for: Transcript Patterns of Bovine CYP21A2 and Its Pseudogene in Adrenal and Ovarian Tissues
Source: Genes (Basel). 2025 Nov 11;16(11):1374. doi: 10.3390/genes16111374 (PMC12652489; doi:10.3390/genes16111374)
Supplement: Supplementary file 1 [file genes-16-01374-s001.zip › genes-3906368-supplementary.pdf]

Supplementary material for :

Transcript patterns of bovine CYP21A2 and its pseudogene in adrenal and ovarian tissues by

Wozniak et al.

Table S1. RNA concentration (ng/µl) and purity (A260/280nm) of adrenal gland and ovarian samples.

**Tissue**	**Samples**	**Concentration (ng/µl)**	**Purity (260/280 nm)**
Adrenal glands	1	173	2.04
	2	814	2.10
	3	495	2.11
	4	597	2.08
	5	498	2.11
	6	826	2.05
Ovaries	1	58	2.05
	2	178	2.09
	3	385	2.12
	4	320	2.12
	5	238	2.11
	6	99	2.08

Table S2. Primers and probes used in qPCR and Sanger sequencing.

**Target gene**	**Primer names**	**Primer sequence 5'→ 3'**	**Amplicon size (bp)**	**Annealing temp. (°C)**
**Real-time PCR**				
CYP21A2	Pair 1	F: AGAGGACCATTGAGGAGGCT	113/222	62
		R: GTCCCCCAGAGAGATGTCCT		
	Pair 2	F: AAGACCTGGGACCACTGGT	130/232	62
		R: TCAGCTGCTTCTCCACCATG		
TATA BOX	TATA_BOX	F: AGCATCTGGCAGTTTCTCAG	148	62
		R: GGGTAAGGGCAACCTAATTT		
**Sanger sequencing of cDNA**				
CYP21A2	Pair 3	F: AGGACCTGAGGGAAGGAGTC	559/667/762	63
		R: CGCAGAACTCCTGGGTCAG		

Table S3. Densitometric values of *CYP21A2* amplicons obtained by semi-quantitative PCR. Background-corrected integrated density (Adj. Total Band Volume) is shown for each band amplified with primer pair 1 or 2 and reference TATA_BOX from adrenal gland and ovarian tissue.

**Primer pair**	**Tissue and amplicon size**	**Adj. Total Band Volume**
Pair 1	Adrenal glands – 113bp	1883750
		1679496
		1872520
		1674234
		1774220
		1815986
	Ovaries – 113bp	1427650
		1415316
		1574304
		1566720
		1636596
		1700300
Pair 2	Adrenal glands – 130bp	2070679
		1793000
		1882538
		2283118
		2354160
		2327722
	Ovaries – 130bp	844580
		1182384
		1033508
		1124864
		1028880
		1130800
	Ovaries – 232bp	372045
		205000
		277928
		105400
		146250
		323850
TATA_BOX	Adrenal glands – 148bp	1030691
		739653
		1042622
		656449
		1438353
		1208904
	Ovaries – 148bp	1263762
		1463434
		1772000
		1929424
		1952993
		1910950

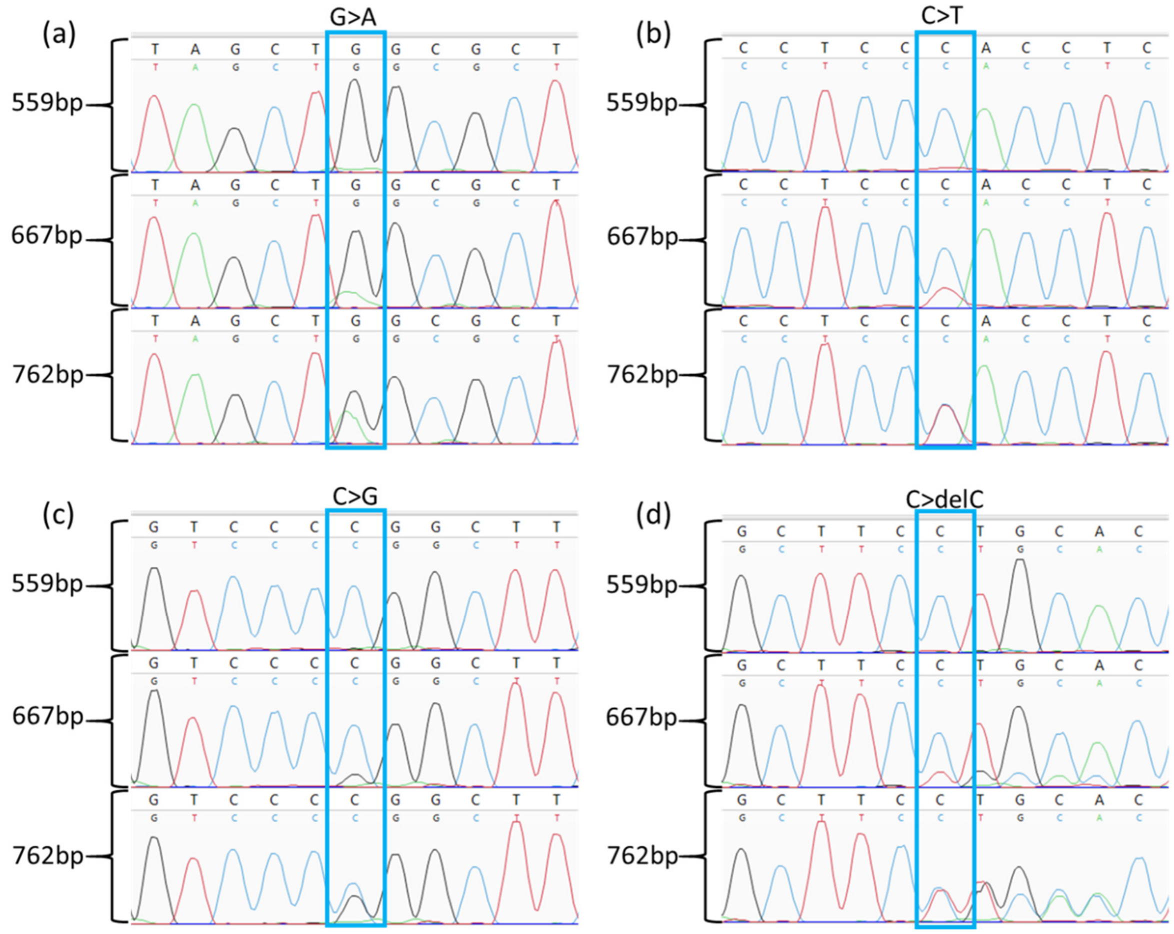

Figure S1. Sanger sequencing chromatograms of cDNA-derived PCR products (559 bp, 667 bp, and 762 bp) for four identified variant positions (a–d). Each panel (a–d) corresponds to a distinct variant and shows chromatograms for the 559 bp product (top), 667 bp product (middle), and 762 bp product (bottom). Blue rectangles indicate the positions of the analyzed variants. In the 667 bp and 762 bp products, secondary or mixed peaks are visible at several positions, reflecting sequence similarity with the *CYP21A1P* pseudogene.

```
      gene GTGGAGAGCTATAAGTGGCGGGGCCGGGGCCTCTGCCTGGGTCTCCAGCCATGGTCCTCG
           |||||||||| |||||||||||||||||||||||||||||||||||||||||||||||||
pseudogene GTGGAGAGCTGTAAGTGGCGGGGCCGGGGCCTCTGCCTGGGTCTCCAGCCATGGTCCTCG

      gene CAGGGCTGCTGCTGCTGCTCACCCTGCTAGCTGGCGCTCACCTGCTATGGGGCCGGTGGA
           |||||||||||||||||||||||||||||||| |||||||||||||||||||||||||||
pseudogene CAGGGCTGCTGCTGCTGCTCACCCTGCTAGCTAGCGCTCACCTGCTATGGGGCCGGTGGA

      gene AGCTCAGAAACCTCCACCTCCCACCTCTGGTCCCCGGCTTCCTGCACCTGCTGCAGCCCA
           ||||||||||||||||||||| |||||||||||| |||||| ||||||||||||||||||
pseudogene AGCTCAGAAACCTCCACCTCCTACCTCTGGTCCCGGGCTTC-TGCACCTGCTGCAGCCCA

      gene ACCTCCCCATCCATCTGCTGAGCCTGACTCAGAAACTCGGGCCTGTCTACAGGCTTCGCC
           ||||||||||||||||||||||||||||||||||||||||||||||||||||||||||||
pseudogene ACCTCCCCATCCATCTGCTGAGCCTGACTCAGAAACTCGGGCCTGTCTACAGGCTTCGCC
```

Figure S2. Alignment of a partial exon 1 sequence of the *CYP21A2* and corresponding pseudogene fragment with 4 differences found in this study (indicated by red arrows).
